# Advances in Wildlife and Exotic Animals’ Anatomy

**DOI:** 10.3390/ani15091208

**Published:** 2025-04-24

**Authors:** María del Mar Yllera, Matilde Lombardero

**Affiliations:** Unit of Veterinary Anatomy and Embryology, Department of Anatomy, Animal Production and Clinical Veterinary Sciences, Faculty of Veterinary Sciences, Terra Campus, University of Santiago de Compostela, 27002 Lugo, Spain; mar.yllera@usc.es

This Special Issue follows a previous one entitled “Advances in Animal Anatomy”. In this editorial, we discuss the significant reduction in the number of hours allocated to the teaching of basic sciences in university curricula. Additionally, undergraduate veterinary programs have traditionally focused on domestic animals. However, many professionals now work with wild or exotic species in zoos, conservation reserves, or veterinary clinics, where these animals have become pets. Veterinarians, researchers, and specialized technicians often lack familiarity with these wild species and those known as new companion animals (NCAs). Thus, in a first contact, and in the absence of specific information about their anatomy, physiology, and pathologies, it is common to consider these animals similar to domestic species in these aspects. Nevertheless, most species have developed unique anatomical and physiological adaptations that can influence their behavior, pathologies, and specific needs.

In the introduction to this issue, we invited experts and researchers to submit articles detailing the particularities of wild species that could assist various professionals working with them. The response has been highly satisfactory.

This Special Issue compiles twenty-four applied anatomy research articles focused on wild species, some of which, due to their peculiar behavior and social nature, have also been introduced into homes, becoming new companion animals. Many of these studies employ modern imaging techniques, often complemented by traditional methods such as macroscopic anatomical dissections, histological preparations, and anatomical sections to identify organic structures (Figure 1). Knowledge of anatomy is fundamental to interpreting the obtained results. Accordingly, six articles utilized computed tomography, another six employed magnetic resonance imaging, one used arthroscopy (rhinoscopy), another conducted histochemical and immunohistochemical studies, and two manuscripts incorporated electron microscopy, in addition to traditional methods such as morphometry, cross-sections, and macroscopic dissections.

Sixteen manuscripts examined wild mammals, occasionally comparing their anatomy with that of domestic species, while four focused on birds, and the remaining four on reptiles.

This Special Issue includes four research articles on carnivorous mammals. Hadžiomerović et al. [Contribution 1] conducted a geometric and morphometric analysis of the auditory ossicles in the red fox (*Vulpes vulpes*). Their research indicated that the anatomical organization of the ossicles was similar, in terms of morphometric parameters, to that described in other carnivores. However, the small sample size of nine animals (seven females and two males) precluded the determination of sexual dimorphism parameters.

The second research article on carnivorous mammals examines the nasal cavity of three large cats: the leopard (*Panthera pardus kotiya* L.), the lion (*Panthera leo leo* L.), and the cheetah (*Acinonyx jubatus jubatus* S.), comparing them to the domestic cat. Although there is some literature on the nasal cavity of the domestic cat using diagnostic imaging techniques such as computed tomography (CT), magnetic resonance imaging (MRI), and rhinoscopy, none is as comprehensive as the study conducted by Díaz Martínez et al. [Contribution 2]. Regarding large cats, there were no previous studies on the anatomy of their nasal cavity using imaging techniques. Transverse, parasagittal, and horizontal images obtained with CT and MRI, along with rhinoscopy images, allowed visualization of the arrangement of the bones, cartilage, and mucosa of the nasal cavity in each species. This information will assist veterinary clinicians in diagnosing pathologies affecting this region, which are quite common in cats.

Vélez García et al. [Contribution 3] investigated the brachial plexus of two procyonids, *Procyon cancrivorus* and *Nasua nasua*, focusing on both the origin and distribution of the nerves derived from it. Meticulous dissections of formalin-preserved specimens were performed, tracing each nerve from its origin to its termination along the thoracic limb to the hand. In both species, the brachial plexus originates from the C5-T2 nerve roots, although variations were observed as C5 and T2 were not constant. The course and distribution of the plexus-derived nerves were similar in all specimens. However, the authors identified specific differences that could be associated with the distinct behaviors of *P. cancrivorus* and *N. nasua*.

Kato et al. [Contribution 4] published the latest paper focusing on a carnivorous mammal, the raccoon (*Procyon lotor*), recently introduced in Japan. After collecting the carcasses, the researchers determined their gender and age, the latter by examining the teeth. Individuals under five months were excluded. Body weight and length were measured. A total of 206 animals were collected and divided into three age groups: juveniles, yearlings, and adults, separated by sex and region of origin. The correlation of body length with sex, age class, and region was tested using multiple regression. Craniometry was performed using digital calipers. The study examines the relationships between craniometrics and body measurements with sex, age class, body mass index, length, and region using a regression model. The researchers also compared the body length and craniometry of their specimens with those described for North American subspecies. Due to inconsistencies in the relationship, the authors suggested that hybridization occurred prior to the introduction of the raccoon in Japan.

This Special Issue includes three publications on rodents: one utilized imaging techniques to describe the anatomy of the porcupine’s head, while the other two examined the digestive tract of the European beaver and the Brazilian agouti from different perspectives.

Morales-Bordon et al. [Contribution 5] combined anatomical sections with magnetic resonance imaging (MRI) to study the skull of the crested porcupine (*Hystrix cristata*) for the first time. Three carcasses were used in this research. After acquiring transverse plane MRI images, two specimens were frozen and cut into transverse slices from the olfactory bulb to the first cervical vertebra. The third specimen was used to obtain transverse, sagittal, and dorsal planes with MRI. By comparing the sections with the MRI images, the researchers were able to identify and describe in detail the cranial structures of the porcupine. This information is an invaluable tool for veterinarians, aiding in the identification of possible pathologies such as fractures, tumors, and abscesses, among others.

The European beaver is the largest rodent in Eurasia. As an herbivorous species, its diet varies with the seasons. Previous studies have indicated that the structure of the esophagus differs between species, and is related, among other factors, to the physicochemical properties of food. Martyniuk et al. [Contribution 6] examined the histology and ultrastructure of the esophagus in 18 adult animals and three fetuses. Their findings revealed that the mucosa, which lacks glands, was lined with stratified squamous keratinized epithelium in both adults and fetuses, and it was thicker in winter, when the animals consumed large quantities of woody plants. Immunohistochemical studies demonstrated a high rate of epithelial proliferation in winter, higher than in spring and summer. No seasonal differences were observed in the other layers of the esophagus.

Jones et al. [Contribution 7] conducted a similar study on the orange-rumped agouti (*Dasyprocta leporine*, Linnaeus, 1758). Observations of this species in the wild led to its classification as omnivorous due to its behavior. A gross anatomical, histologic, morphometric, and histometric analysis of the digestive tract was performed on six adult animals. Their esophagus was also lined with keratinized epithelium, an adaptation to roughage feeding, and esophageal glands were present. The simple stomach, with abundant gastric glands, was typical of carnivorous species, while the large development of the caecum and colon corresponded to a frugivorous and herbivorous diet. The histology of the different sections of the gastrointestinal tract also supported this conclusion.

Ticks can serve as vectors for multiple pathogens. They feed on blood and climb onto a host’s hair coat from their habitat. Interestingly, they do not attack all animals equally; some species appear to be more attractive or sensitive to them than others. Lee et al. [Contribution 8] studied the skin characteristics of two deer species, the Korean water deer (*Hydropotes inermis argyropus*) and the roe deer (*Capreolus pygargus*), to understand why the former had a much lower tick infestation rate than the latter. They collected tissue samples from five individuals of each species, counted their number of ticks, and conducted microscopic studies of the hair and skin. As expected, their results confirmed a much lower infestation rate in the Korean water deer. The article also describes significant differences in hair properties, including diameter and density. The hair of the Korean water deer was thicker and its density much higher, which could hinder the movement and feeding of ticks. The skin of the Korean water deer had mainly primary follicles, while the skin of the roe deer was dominated by a pattern composed of primary and secondary follicles. The authors suggested that this difference could create environments less favorable for ticks in the former case.

The rostral epidural rete mirabile (*rete mirabile epidurale rostrale*) is a structure exclusively described in mammals of the order Artiodactyla. It comprises a network of small anastomosed arterial vessels located at the base of the brain on either side of the pituitary gland. Its primary function is to maintain a constant brain temperature. The arterial blood supplied to this *rete* may, in certain instances, be warmer than the organ itself. Situated within the cavernous sinus, whose blood has been cooled in the nasal cavity, the rete facilitates heat transfer from the arterial to the venous blood, thereby equalizing their temperatures. The presence of the rostral epidural rete mirabile is characteristic of nearly all animals within the Artiodactyla order. Zdun [Contribution 9] investigated its morphology in some wild Suiformes and hippopotamuses by injecting a red solution of the chemo-setting acrylic material Duracryl^®^ Plus into both common carotid arteries and/or a blue solution into the bilateral external jugular veins. Some specimens were subjected to enzymatic digestion of soft tissues, while others were fixed in 5% formalin for dissection. They observed that the rostral branch to the rostral epidural rete mirabile is a single vessel in Suiformes, whereas in hippopotamuses, there were three to five vessels.

Three articles focusing on marsupials are included in this Special Issue. Zdun et al. [Contribution 10] described, for the first time, the arterial blood supply to the brain of the red-necked wallaby (*Notamacropus rufogriseus*). Using the same methods as in their previous manuscript, and incorporating an angio-CT examination, they obtained detailed information about the brain vascularization of this species. The internal carotid artery was identified as the main vessel supplying blood to the brain, with no carotid sinus present. This vessel entered the cranial cavity through the optic canal, and branched into smaller vessels. In the red-necked wallaby, Zdun et al. [Contribution 10] observed that the arterial circle of the brain was closed from the caudal side. The authors compared the cerebral anatomy of this species with that described in other mammalian groups.

Yllera et al. [Contributions 11,12] focused their studies on the sugar glider, a small Australian marsupial that is becoming increasingly popular as a companion animal. Given the unique reproductive characteristics of marsupials, they examined both the female [Yllera et al., Contribution 11] and male [Yllera et al., Contribution 12] genital apparatus through meticulous anatomical dissections, including some microscopic insights in males. The females exhibited duplicated ovaries, oviducts, uteri, and vaginas. During pregnancy, a third vagina, known as the birth canal, forms to facilitate the emergence of the offspring. In males, the reproductive system, similar to that of other marsupials, closely resembled that of placental mammals. It included a pair of testicles, two epididymides, two deferent ducts, the urethra, a bifid penis, and two types of accessory glands: the prostate and two pairs of bulbourethral glands. In this species, the genital, urinary, and digestive systems converge into a cavity called the cloaca, which communicates with the exterior. These articles provide detailed and valuable information to veterinary clinicians, aiding in accurate diagnosis, interpretation of modern imaging techniques such as MRI and CT, and successful surgical interventions in the genital tract when necessary.

Marine mammals constitute a diverse group of animals that have adapted to life in the sea or rely on the sea for sustenance. Many populations are vulnerable or endangered due to a long history of commercial exploitation, habitat degradation, or pollution. Currently, significant efforts are being made to halt their decline and promote their recovery. However, a comprehensive understanding of their habitat, behavior, feeding, reproduction, physiology, and anatomy is essential. Consequently, the study of marine mammals is one of the most active fields of research.

Three articles in this Special Issue focus on the anatomy of the Franciscana dolphin (*Pontoporia blainvillei*) and the South American fur seal (*Arctocephalus australis*). It is widely accepted that anatomy is crucial for understanding many physiological adaptations. Therefore, these data can be instrumental in developing effective conservation plans, and provide essential knowledge for clinical veterinarians who treat pathologies in marine mammals, both in the wild and in captivity.

Tostado-Marcos et al. [Contribution 13] conducted meticulous anatomical dissections of 11 adult and juvenile dolphins, examining their digestive tracts and comparing them with those of other marine and terrestrial carnivores. They identified several peculiarities in the tongue, teeth, stomach, and small intestine, suggesting that the Franciscana dolphin’s digestive system has adapted to a diet consisting of small prey.

Martín-Orti et al. [Contribution 14] and Molpeceres-Diego et al. [Contribution 15] investigated the South American fur seal, focusing on the digestive and respiratory systems, respectively. Both studies primarily employed anatomical dissection, supplemented by radiological analysis of the main digestive viscera in the former, and by inflating the lungs with compressed air and creating a polyurethane cast of the entire bronchial tree in the latter. Both articles compared the anatomy of the fur seal with that of terrestrial carnivores, particularly the dog. The authors identified significant similarities between the two species, although the seal exhibited some specific characteristics. These differences were suggested to be adaptations to their feeding habits and marine environment, respectively.

Five manuscripts focused on seabirds, including two on Cory’s Shearwater (*Calonectris borealis*) and three on the Atlantic puffin (*Fratercula arctica*), are included in this Special Issue. It is well known that significant anatomical differences exist between birds and mammals. Among birds, seabirds possess unique characteristics due to their adaptation to various environments: aquatic, terrestrial, and aerial. Some seabirds are classified as vulnerable species, necessitating conservation programs, while others are kept in zoos and wildlife reserves or are rescued from the wild when sick or injured, requiring veterinary care. Often, professionals (veterinarians, biologists) responsible for their care or recovery plans lack specific data on the species in question, and must adapt information from closely related species. These articles partially describe their anatomy and employ modern diagnostic imaging methods, providing knowledge that could be highly useful in aiding the conservation of seabirds.

Morales-Espino et al. investigated the coelomic cavity [Contribution 16] and the head [Contribution 17] of Cory’s Shearwater using anatomical cross-sections, computed tomography (CT), and magnetic resonance imaging (MRI). Birds lack a diaphragm, resulting in a single body cavity: the coelomic cavity. The authors combined dissections of fresh cadavers with CT images and anatomical cross-sections of frozen specimens to identify and determine the spatial organization of the various organs within the body cavity. In their second study [Morales-Espino et al., Contribution 17], the authors focused on the anatomy of the bird’s head. By combining transverse and dorsal anatomical sections of frozen specimens with sequential transverse CT images and MRI sequences in the transverse plane, they described the detailed anatomy of Cory’s Shearwater’s head, including the central nervous system and its associated structures.

Jaber et al. [Contribution 18] and Fumero-Hernández et al. [Contributions 19,20] conducted analogous studies utilizing cross-sectional anatomy and CT or MRI on the coelomic cavity [Jaber et al., Contribution 18], head [Fumero-Hernández et al., Contribution 19], and eyeball [Fumero-Hernández et al., Contribution 20] of the Atlantic puffin (*Fratercula arctica).* Additionally, Jaber et al. [Contribution 18] performed dissections on three specimens to visualize the organ locations within the coelomic cavity. These dissections enabled the precise identification and correlation of anatomical features observed in anatomical sections and CT images.

Birds possess one of the most acute senses of sight in the animal kingdom. Their eyes contain anatomical structures absent in mammals, such as the pecten and the scleral ring. Fumero-Hernández et al. [Contribution 20] obtained CT images in various planes of the puffin’s head, concentrating on the ocular structures. The morphometric study results of these anatomical elements provided significant information that could aid in interpreting the behavioral patterns of this species.

Turning to reptiles, this Special Issue includes four studies. The first study by Kandyel et al. [Contribution 21] focuses on the Egyptian bridled skink (*Heremites vittatus*). Gross anatomy, electron microscopy, histochemistry, and immunohistochemistry were employed to examine the morphological characteristics of the tongue and laryngeal mound. The tongue of this species is triangular in shape, and is divided into three zones: foretongue, midtongue, and hindtongue. The authors identified nine subtypes of filiform papillae with a specific distribution on the dorsal surface of the tongue, while the ventral apical surface of the foretongue comprised conical papillae. Taste pores and taste buds were observed on the dorsal surface of the foretongue and midtongue, respectively. Histological studies revealed a keratinized layer only on the dorsal surface of the foretongue, whereas lingual glands were present only in the midtongue and hindtongue. The researchers noted a strong expression of cytokeratin throughout the tongue. The authors associated these tongue characteristics with the animal’s feeding behavior.

González Rodríguez et al. [Contribution 22] utilized macroscopic cross-sections of frozen specimens in conjunction with modern imaging techniques (CT and MRI) to identify the primary structures of the rhinoceros iguana head (*Cyclura cornuta cornuta*), a reptile classified as vulnerable by the International Union for Conservation of Nature (IUCN). The limited literature available on this species primarily focuses on genetic and evolutionary studies or descriptions of pathological cases. Therefore, the anatomical information presented in this article, encompassing both soft tissues and bones, could be highly valuable for diagnosing pathologies such as fractures, skull malformations, or neoplasia. Additionally, it could serve as a useful tool for recognizing diseases resulting from an unbalanced diet, such as osteodystrophy.

Mohamad et al. [Contribution 23] examined the skulls of two reptile species, the loggerhead turtle (*Caretta caretta*) and the green iguana (*Iguana iguana*), using computed tomography (CT) reconstructive techniques such as maximum intensity projections (MIP) and volume rendering (VR). They studied five adult female turtles and four adult male iguanas, taking various measurements of their skulls. Transverse CT images were also obtained, and the data were transferred to a CT workstation to generate VR and MIP reconstructed images of the head. Three-dimensional reconstructions facilitated the visualization of cranial bones. Consequently, Mohamad et al. [Contribution 23] described the skull bones in both species, and highlighted the most significant anatomical differences between them. They concluded that these reconstruction techniques could be highly useful not only for diagnosing various pathological conditions, but also for teaching veterinary anatomy with realistic images.

The latest article on reptiles included in this Special Issue focused on the eyeball of the loggerhead turtle (*Caretta caretta*). This species spends most of its life at sea, necessitating certain adaptations, including well-developed nictitating membranes and sclerotic rings to protect the eye. Fumero-Hernández et al. [Contribution 24] conducted a morphometric study of the loggerhead turtle’s eyeball using computed tomography. They studied 10 live animals and measured the length of their shells. The authors also took various measurements of different elements of the eyeball (*n* = 20), as well as the scleral ring, using oblique sagittal, transverse, and dorsal CT images of the skulls, and performed statistical analysis. Consequently, they obtained reference values for the eyeball and scleral ring. The authors suggested that these values could be related to the vision capabilities and habits of these animals. They acknowledged two limitations to their study: the low number of animals used and their varying sizes, and the inherent error associated with the manual contouring of computed tomography images.

We believe that this Special Issue has successfully met our objective of assisting various professionals (veterinarians, biologists, keepers, and technicians) who work with wild animals, both in their natural habitats and in captivity, by providing them with new anatomical knowledge and aiding in the interpretation of results obtained through modern diagnostic imaging techniques. We acknowledge that these contributions are on a limited number of species, but we hope they will positively impact on the care and welfare of wild animals. In this manner, we aim to contribute to the conservation of biodiversity.

## Figures and Tables

**Figure 1:**
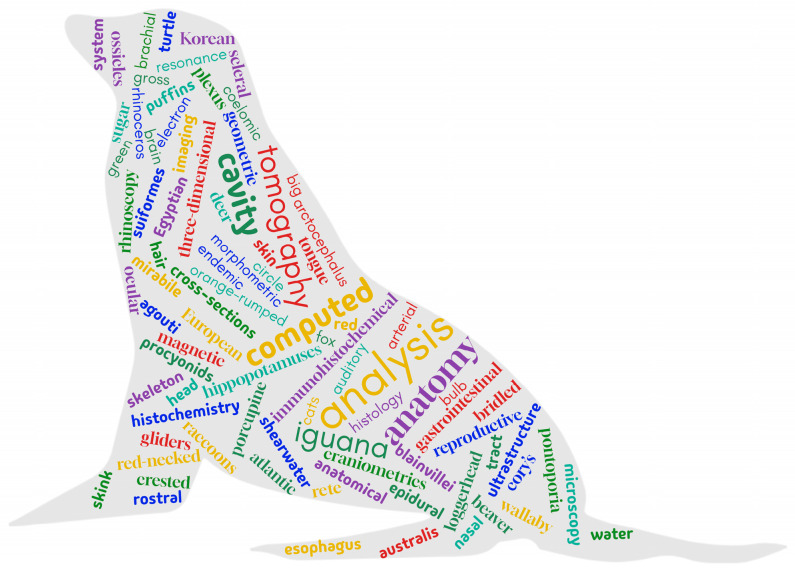
A word cloud featuring keywords from the articles included in this Special Issue.

